# Multifocal Tuberculosis Verrucosa Cutis: Case Report and Review of the Literature

**DOI:** 10.3390/medicina59101758

**Published:** 2023-10-02

**Authors:** Niki Ntavari, Vasiliki Syrmou, Konstantinos Tourlakopoulos, Foteini Malli, Irini Gerogianni, Angeliki-Viktoria Roussaki, Efterpi Zafiriou, Maria Ioannou, Eirini Tziastoudi, Konstantinos I. Gourgoulianis, Ioannis Pantazopoulos

**Affiliations:** 1 Department of Dermatology, Faculty of Medicine, University of Thessaly, 41500 Larissa, Greece; nikintavari@gmail.com (N.N.); roussaki@med.uth.gr (A.-V.R.); zafevi@o365.uth.gr (E.Z.); 2 Department of Rheumatology and Clinical Immunology, University of Thessaly, 41500 Larissa, Greece; syrmouvicky@yahoo.gr; 3 Department of Respiratory Medicine, Faculty of Medicine, University of Thessaly, 41500 Larissa, Greece; kntourlakopoulos@gmail.com (K.T.); igerogianni@yahoo.gr (I.G.); kgourg@med.uth.gr (K.I.G.); pantazopoulosioannis@yahoo.com (I.P.); 4 Respiratory Disorders Lab, Faculty of Nursing, University of Thessaly, 41500 Larissa, Greece; 5 Department of Pathology, Faculty of Medicine, University of Thessaly, 41334 Larissa, Greece; mioan@med.uth.gr (M.I.); etziastoudi@gmail.com (E.T.); 6 Department of Emergency Medicine, Faculty of Medicine, University of Thessaly, 41334 Larissa, Greece

**Keywords:** tuberculosis verrucosa cutis (TBVC), multifocal, infectious diseases, tuberculosis

## Abstract

Cutaneous tuberculosis (TB) is still a major public health problem worldwide. Tuberculosis verrucosa cutis (TBVC) is a cutaneous form of exogenous TB caused by exogenous reinfection in previously sensitized individuals. TBVC typically presents as a unifocal condition. Multifocal cutaneous lesions without any other tubercular foci are extremely rare in exogenous TB and few cases are reported in the literature. We describe the first case of multifocal TBVC in an 81-year-old Greek man. In total, 14 cases of multifocal TBVC have been reported in the literature (8 males and 6 females), with mean age 47.64 years (SD = 20.75) and mean time to diagnosis of 9.69 years (SD = 15.31). Most cases (11/12) responded rapidly to treatment, implying the accuracy of diagnosis, while no one was reported to be immunocompromised. Finally, in 10 cases (71.4%), history of skin microtrauma was reported (related either to daily life habits or to professional praxis), confirming it as the main risk factor. The tuberculin skin test was positive in 10 cases and tissue culture for mycobacteria was negative in all cases. TBVC can present with multiple lesions, even in countries where TB prevalence is not high, especially in patients with history of skin abrasions. Prompt specialist assessment can expedite the establishment of diagnosis.

## 1. Introduction

Cutaneous tuberculosis (TB) is frequently seen among dermatology outpatient departments in India [1]. On the other hand, it is uncommon in developed countries, although it has an increasing incidence among minorities of Indian and African origin. Cutaneous TB can be acquired from hematogenous or lymphatic dissemination of a pulmonary focus, being in many cases multifocal (endogenous cutaneous TB) and otherwise arising through direct inoculation with a more local predominance (exogenous cutaneous TB) [2]. Tuberculosis verrucosa cutis (TBVC) represents the most common form of exogenous TB. Multifocal cutaneous lesions without any other tubercular focus are extremely rare in exogenous TB.

Herein, we report a case of multifocal TBVC presenting with different types of skin rashes depending on the affected site, obstructing easy diagnosis of cutaneous TB. Furthermore, we summarize and compare the available literature on multifocal TBVC. Written and informed permission for publication was obtained from the patient.

## 2. Detailed Case Description

An 81-year-old, male, a former lawyer, was referred to our outpatient dermatology department with a 4-month-long, polymorphic, multifocal skin rash on the scalp, back, and upper and lower limbs. The patient complained of intense pruritus on the scalp and lower limb pain upon the lesions. He did not report a cough, hemoptysis, fever, night sweats, or weight loss. There was no history of smoking or alcohol abuse. He was not aware of preceding TB infection, and he could not recall any exposure to a known TB case. In terms of past medical history, he reported insulin-dependent type 2 diabetes mellitus, percutaneous transluminal coronary angioplasty, atrial fibrillation, and angiodysplasia of the gastrointestinal tract. He had been vaccinated with the Bacille Calmette–Guerin (BCG) vaccine at the age of 18.

**Figure 1:**
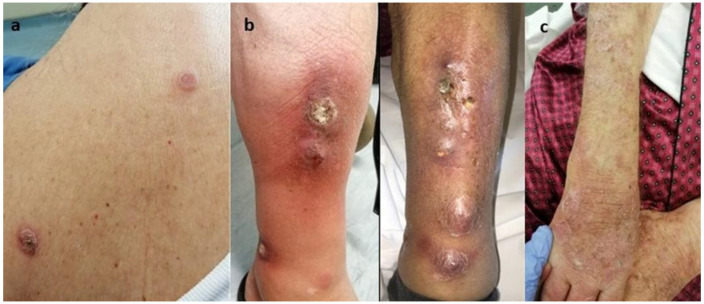
(**a**) Erythematous papules and small plaques with an arciform pattern in the patients’ back, (**b**) erythematous, subcutaneous nodules and small scaly plaques in the calves, (**c**) rash on the upper limbs.

On physical examination, multiple crusted, scaly lesions with marginal erythema and erosions were observed on the scalp. He reported that the lesions were present for several months, but he noticed a sharp growth and the presence of erosions during the last month. A second rash was observed on the patient’s back and was characterized by tender, erythematous papules, and small plaques with an arciform pattern, similar to those of Sweet Syndrome (Figure 1a). One of the lesions demonstrated central crusting. In addition, painful, erythematous, subcutaneous nodules with a diameter of 1–2 cm and small scaly plaques were palpable on the patient’s lower limbs. The patient mentioned that they were present for a 4-month period (Figure 1b). The nodules were located on the anterior aspect of the patient’s calves and shins. The lesions started as painless small skin lesions before progressively enlarging and ulcerating. On the left lower limb, the inflammation overlying the nodules merged, developing a large erythematous lesion without ulceration. In contrast, the nodules showed ulceration with mixed purulent/sanguineous discharge. Left ankle swelling was also observed. On the right shin, there were fewer lesions, and more importantly, a larger less painful ulcerated nodule with central crusting. The patient’s most recent rash emerged on the upper limbs 15 days prior to their visit (Figure 1c). No regional or generalized lymphadenopathy was observed. The differential diagnosis included tuberculosis verrucosa cutis, multiple tuberculous abscess, nontuberculous mycobacterial infection, and deep fungal infection.

Routine blood tests revealed leukocytosis. The patient had a total leukocyte count of 17,700 cells/mm³ with a differential count of 70% neutrophils, 3% eosinophils, 10% lymphocytes, and an erythrocyte sedimentation rate of 40 mm in the 1st hour. Biochemistry test results and autoimmune markers (including antinuclear antibodies, p- and c-antineutrophil cytoplasmic antibodies, and anti-proteinase 3 anti-myeloperoxidase antibodies) were unremarkable. Thyroid function tests were normal and serum electrophoresis revealed no monoclonal component. VDRL and HIV tests were non-reactive. Chest X-ray revealed no evidence of tuberculosis or any other abnormality. The tuberculin skin test (TST) (SPAN’s tuberculin) was 20 mm. Although the patient did not present with fever, the possibility of “Sweet Syndrome” was considered and chest and abdominal computed tomography were conducted without any abnormal findings. Examination of pus and skin scraping with Ziehl Nielsen staining failed to identify any acid-fast bacilli. Scraping was performed on the margin of the lesions on the legs and the back, and a pathology slide was prepared with 10% KOH. The result was negative for mycelia/spores.

**Figure 2:**
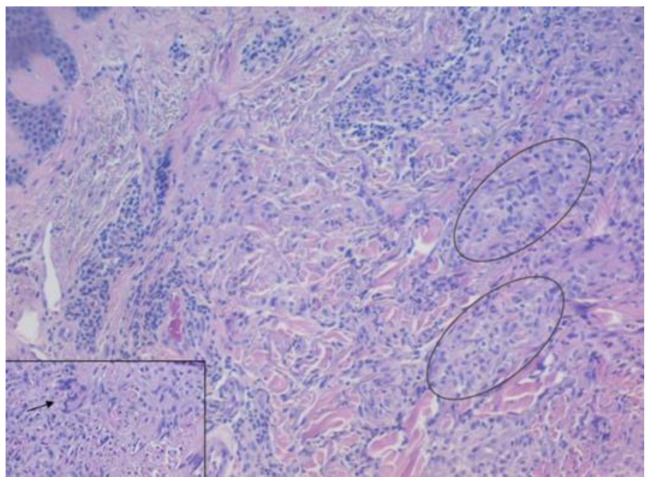
Histology shows a granulomatous inflammation composed of epithelioid histiocytes with sporadic multinucleated giant cells (as present in the rectangle insert) and lymphocytes (hematoxylin and eosin). Hematoxylin and Eosin staining, original magnification ×4. Insert original magnification ×40.

**Figure 3:**
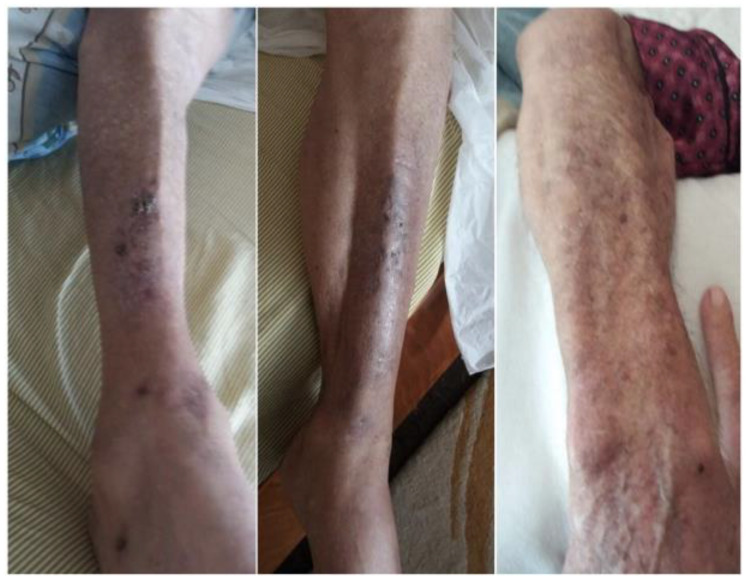
Improvement of the nodules and ulcers after 2 months of antituberculous treatment.

A 4 mm punch biopsy specimen was taken from the lesions of the back and upper and lower limbs. Histopathology revealed hyperkeratosis, acanthosis with pseudoepitheliomatous hyperplasia of the epidermis, and diffuse lymphocytic and neutrophilic inflammatory infiltrates (Figure 2). Tuberculoid granulomatous inflammation in the dermis was also observed. A culture for mycobacterium TB was negative. Periodic acid Schiff staining did not reveal fungal elements. Based on the above-mentioned histology findings, the diagnosis of TBVC was made and the patient was started on a six-month antitubercular regimen with rifampicin, isoniazid, pyrazinamide, and ethambutol. On a follow-up visit 2 months later, the nodules had flattened out and the ulcers had disappeared (Figure 3).

## 3. Discussion

Cutaneous tuberculosis spans a spectrum, ranging from lupus vulgaris and TBVC at one end to scrofuloderma and tuberculosis cutis orificialis at the other end [3]. The progression along this spectrum is associated with a decline in cell-mediated immunity [3]. TBVC can be acquired via exogenous direct skin inoculation of M. tuberculosis and M. bovis through abrasions in a previously sensitized patient with a moderate to high degree of immunity to mycobacterium TB infection [4]. Thus, it is found predominately in anatomical regions prone to trauma such as the soles, the dorsum of the hands and feet, the fingers, and the toes [2]. Typically, this form of cutaneous TB is observed as a single lesion in one anatomical site. Multifocal TBVC is quite rare and, in our literature search, only 13 published cases were identified (Table 1). Herein, we report the first case of multifocal TBVC in a Greek man, where multiple lesions were identified in the scalp, back, and upper and lower limbs. In our patient, chronic generalized pruritus, most possibly related to type 2 diabetes mellitus, is believed to have been the cause of itching, leading to microtrauma to the skin. Moreover, the patient was self-injecting with insulin and was measuring his glucose blood capillary level at least twice daily, causing micro-needle injuries on the fingertips and abdominal wall.

In Greece, the BCG vaccine was included in the national vaccination schedule until 2016 and this patient was vaccinated. Despite vaccination, he had probably been infected by and successfully cleared the bacillus during his lifetime, acquiring a stronger degree of immunity against the pathogen. This, in turn, at a later phase, led to TBVC, following an exogenous inoculation of the pathogen in the microlesions of the skin. This is not a common phenomenon in Greece; it can, however, be explained by the fact that TB has not been completely eradicated according to authorities. Interestingly, nearly all published cases were noted in Indian patients, except for one patient from the Republic of North Macedonia [5] and one from Bangladesh [6]. Since the development of TBVC requires previous immunity against TB, it is expected to be more common in endemic countries where the prevalence of TB is higher. Moreover, it represents a form of locally secluded bacilli lesion, managed by the body with the formation of a granuloma, which confines the disease to this specific point. For this reason, it is not common to have multiple inoculations in the same person on different body parts. It is noteworthy that although TBVC is reported to be more frequent in children living in endemic countries and walking barefoot on ground contaminated with tuberculous sputum, only two cases of multifocal TBVC have been reported in children aged below 18 years (one in a 17- and one in a 12-year-old child) [6,7].

The long timespan from lesion manifestation till TBVC diagnosis is partially explained by the lack of systemic symptoms that would prompt the patient to seek medical advice early. In countries with paucity of proper primary healthcare services and with the health system prioritizing more severe public health problems, patients with mild skin lesions that do not pose immediate threat to life or do not cause significant disability can go undiagnosed for years. TBVC lesions run an asymptomatic course in most cases and start as small papules that slowly progress to verrucous ones over several months to years later [2]. For this reason, they usually run a prolonged course before being diagnosed. The longest period described in the literature was 60 years, in a 65-year-old patient from the Republic of North Macedonia with multifocal TBVC. The average duration for the diagnosis of multifocal TBVC is nearly 10 years, further indicating the long course of the disease before diagnosis [5]. In our case, the patient had small subcutaneous nodules of 1–2 cm diameter, and small scaly plaques at the lower limbs for a 4-month period. Early referral to a highly specialized unit can explain the timely diagnosis and treatment. Another interesting finding in our case was that not all the lesions appeared with the same macroscopic morphology. According to the other published multifocal TBVC cases, the initial lesion of TBVC appears as a small, painless papule with an inflammatory border progressing to the hyperkeratotic plaque with peripheral extension [2]. The center of the lesion may remain hyperkeratotic, with a white atrophic scar, or may exude pus [2].

TBVC develops in patients with an intact immune system [17], previously exposed and sensitized to the mycobacterium. A robust immune response seems to be a prerequisite for this form of TB. This is expected as the individual should be sensitized against the pathogen, with effective clearance already performed. In these patients, skin reaction to seclude bacilli leads to the typical tuberculoid granuloma formation.

As TBVC is caused by accidental inoculation through open wounds or abrasions, certain professional groups are at higher risk of becoming infected [18]. As evident in Table 1, most patients with multifocal TBVC were farmers, butchers, or cattle ranchers. Interestingly, our patient was a lawyer without any obvious inoculation site, but with history of pruritus, itching, and regular insulin injections. Multifocal TBVC mainly affects the lower limbs, but it can also affect the buttocks, the upper limbs, and even the head, as seen in our case.

The diagnosis of TBVC is challenging and is mainly based on the correlation of the medical and disease course history, the physical findings (i.e., the characteristic lesions, etc.), and evidence of TB infection (i.e., the culture of the biopsy specimens, positive TST, etc.), while the most important diagnostic tool is the histopathological examination [19]. The lesions are characterized by pseudoepitheliomatous hyperplasia of the epidermis with hyperkeratosis and dense inflammatory cell infiltrates comprising of neutrophils, lymphocytes, and Langhans giant cells [19]. Moreover, tuberculous granulomas with caseous necrosis of moderate intensity can be found in the dermis [19]. However, caseous necrosis in the dermis may not always be evident, as seen in some of the cases presented in Table 1.

TST is typically positive, representing a delayed hypersensitivity reaction, engaging CD4+ T cells sensitized by prior infection [20]. However, as seen in our results, there have been cases of multifocal TBVC with negative TST. For this reason, TST should be performed in all cases, but clinicians should not exclude the diagnosis in the event of a negative result. Tissue mycobacterial culture, although helpful in making a diagnosis, is usually negative (paucibacillary form) as a result of the strong immune response against the bacilli [21]. No positive culture for mycobacterium TB has been reported in patients with multifocal TBVC (Table 1). Molecular tests, when available, such as mycobacterial DNA isolation, can confirm the diagnosis. Nucleic acid amplification (NAA) techniques in tissue specimens can be useful as they can provide early evidence of infection (within 2 h) and information regarding genes associated with drug resistance [8]. Regarding paucibacillary forms, the results are controversial [9,10]. However, in cases where tissue culture and histopathologic stains are both negative, they can assist in the establishment of the diagnosis [10].

TBVC is treated as per the recommendations of therapy for pulmonary TB and the therapeutic response represents a valuable diagnostic criterion [11]. Multifocal TBVC responds to antituberculous treatment within three to six months, as observed in most cases mentioned in Table 1. Response to treatment indirectly confirms the diagnosis, as pathogen isolation is not possible in several instances. Only one case reported a minimal response after one year of treatment [12]. Surgical excision, cryotherapy, and electrocautery can be useful in the treatment of localized lesions in addition to systemic treatment.

After an extensive literature review, we identified 13 cases of multifocal TBVC (Table 1). Including our case, eight patients were males and six were females, giving a male-to-female ratio of 1.33. The mean age was 47.64 ± 20.75 years and the mean time from symptom onset to diagnosis was 9.69 ± 15.31 years. In two cases, information was not provided regarding immunosuppression, but all other cases were described in immunocompetent patients. Eleven individuals were of Asian origin (ten from India and one from Bangladesh) and two came from the Balkan peninsula (one from Greece and one from the Republic of North Macedonia). Ten cases had positive TST (76.9%). Tissue culture for mycobacteria was performed in 11 cases and was negative in all cases, while for the other 3 cases, information was unavailable. In terms of treatment outcome, information was not provided for 2 cases, whereas, in the remaining 12 cases, the lesions persisted one year after initiation of treatment only in 1 woman. Most cases (11/12, 91.6%) had a significant response to treatment within six months. Finally, in 10 cases (71.4%), history of skin microtrauma was provided, indicating it as the main risk factor.

Tuberculosis comprises a major burden for public health worldwide despite the tremendous progress in technology, biomedical research, and pharmacology. This phenomenon is believed to exist due to socioeconomic differences, the global financial crisis, public healthcare services’ incompetence, antibiotic resistance, and recently, the SARS-CoV-2 pandemic (delayed diagnosis, limited financial sources, limited access to healthcare services).

## 4. Conclusions

In conclusion, cutaneous TB, as part of the TB spectrum, can still comprise a challenge for clinicians worldwide. Compatible skin lesions and histology should support the decision to initiate treatment. Quick response to treatment provides the most valuable evidence of an accurate diagnosis. The present case report highlights the importance of considering multifocal TBVC even when various types of skin rashes appear at different sites.

## Figures and Tables

**Table 1:** Brief presentation of the published studies on multifocal TBVC.

First Author/Year	Age/Sex	Country	Duration	Immunosuppression/Cause	Site	Culture TB/Mantoux	Histology	Response to Therapy
Damevska K/2013 [4]	65, F	Republic of North Macedonia	60 y	Immunocompetent/Ν.A.	Bilateral upper limbs and right lower limb	Negative/(+)	Granulomatous inflamma-tion in the dermis, with small foci of caseation necrosis	Damevska K/2013 [4]
Rahman MH/2020 [5]	12, M	Bangladesh	2 y	Immunocompetent/bare-footed child	Bilateral extremities	N.A./(+) 17 mm	Hypertrophy of the epidermis and mid-dermalgranuloma with Langhans giant cells	At 6 months, complete resolution of the lesions
Rajan J/2011 [6]	17, M	India	2 y	Immunocompetent/cattleherd	Left foot	N.A./(+) 17 mm	Hypertrophy of the epidermis and mid-dermal granulomata with Langhans giant cells	At 6 months, all the lesions were completely resolved
Vora RV/2016 [7]	60, F	India	12 y	Immunocompetent/a thorn prick over her right big toe	Right lower limb	N.A./(−)	Hyperkeratosis, acanthosis of the epidermis. Below the epidermis, multiple granulomas, comprised epithelioid cells with Langhans	At 3 months, improvement of the lesions
Sudarshan R/2016 [8]	69, F	India	14 y	Immunocompetent/farmer handling cattle occasionally	Right upperlimb, lower limbs, face and nape	Negative/(−)	Hyperplasia, hyperkeratosis and hypergranulosis. Dermis epithelioid cell granulomas with many Langhans giant cells and lymphoplasmacytic infiltrate	Incomplete response after 1 year of therapy
Chahar M/2015 [9]	48, M	India	15 y	Immunocompetent/walking barefoot	Bilateral buttocks and feet	Negative/(+)	Hyperkeratosis, acanthosis, papillomatosis. Upper dermis mononuclear infiltrate and mid-dermis. Epithelioid cell granulomas comprising Langhans giant cells	At 6 months, complete resolution of the lesions
Verma R/2014 [10]	30, M	India	3 y	Immunocompetent/farmer	Right lower limb	Negative/(+)	Pseudoepitheliomatous hyperplasia with irregular acanthosis. Dermis epithelioid cell granulomas with Langhans giant cells and neutrophilic microabscesses	At 6 months, resolution of the lesions
Rasineni N/2014 [11]	42, F	India	4 m	N.A./N.A.	Left sole and left index finger	Negative/(+) 20 mm	Hyperkeratosis along with numerous lymphocytes, epithelioid cells, and Langhans giant cells in the dermis	At 5 months, complete clearance
Manjumeena D/2018 [12]	52, F	India	20 y	Immunocompetent/trauma while cutting wood	Left leg and foot	Negative/(+)	Hyperkeratosis, acanthosis, papillomatosis, granulomas composed of lymphocytes, neutrophils, giant cells with central caseous necrosis	At 6 months, regression of the lesions
Sehgal VN/2017 [13]	78, M	India	1 y	N.A/N.A	upper and lower extremities	N.A./(−)	Epithelial hyperplasia, papillomatosis, and perivascular inflammation in dermis. Epithelioid cell granuloma	At 6 months, complete regression of the lesions
Rani S/2020 [14]	29, M	India	2 y	Immunocompetent/farmer	Heel, antero-posterior and medial side of leg	Negative/(+) 20 mm	Hyperkeratosis, acanthosis, dermal infiltration with epithelioid cell granulomas with Langhans giant cells and occasional central necrosis	At 5 months, improvement of the lesions
Prasad PVS/2002 [15]	35, M	India	2 y	Immunocompetent	Left hand and left foot	Negative/(−)	Hyperkeratosis, acanthosis, and mid-dermal tuberculoid granulomas	-
Padmaprasad MK/2013 [16]	49, F	India	2 y	Immunocompetent/butcher	Right shoulder, right breast, lower limbs and buttocks	Negative/(+) 22 mm	Pseudoepitheliomatous hyperplasia, dense infiltration of plasma cells and giant cells, and caseation necrosis	N.A.

## Data Availability

Not applicable.

## References

[1] Kumar B., Kaur S. (1986). Pattern of Cutaneous Tuberculosis in North India. Indian J. Dermatol. Venereol. Leprol..

[2] Santos J.B.D., Figueiredo A.R., Ferraz C.E., de Oliveira M.H., da Silva P.G., de Medeiros V.L.S. (2014). Cutaneous tuberculosis: Epidemiologic, etiopathogenic and clinical aspects—Part I. An. Bras. Dermatol..

[3] Charifa A., Mangat R., Oakley A.M. (2023). Cutaneous Tuberculosis.

[4] Sehgal V.N., Sehgal R., Bajaj P., Sriviastava G., Bhattacharya S. (2000). Tuberculosis verrucosa cutis (TBVC). J. Eur. Acad. Dermatol. Venereol. JEADV.

[5] Damevska K., Gocev G. (2013). Multifocal tuberculosis verrucosa cutis of 60 years duration. Int. J. Infect. Dis. IJID Off. Publ. Int. Soc. Infect. Dis..

[6] Rahman M.H., Ansari N.P. (2011). Extensive multifocal tuberculosis verrucosa cutis in a young child. Med. Pract. Rev..

[7] Rajan J., Mathai A.T., Prasad P.V.S., Kaviarasan P.K. (2011). Multifocal tuberculosis verrucosa cutis. Indian J. Dermatol..

[8] Acharya B., Acharya A., Gautam S., Ghimire S.P., Mishra G., Parajuli N., Sapkota B. (2020). Advances in diagnosis of Tuberculosis: An update into molecular diagnosis of Mycobacterium tuberculosis. Mol. Biol. Rep..

[9] Tan S.H., Tan B.H., Goh C.L., Tan K.C., Tan M.F., Ng W.C., Tan W.C. (1999). Detection of Mycobacterium tuberculosis DNA using polymerase chain reaction in cutaneous tuberculosis and tuberculids. Int. J. Dermatol..

[10] Hsiao P.-F., Tzen C.-Y., Chen H.-C., Su H.-Y. (2003). Polymerase chain reaction based detection of Mycobacterium tuberculosis in tissues showing granulomatous inflammation without demonstrable acid-fast bacilli. Int. J. Dermatol..

[11] Pebriany D., Anwar A.I., Djamaludin W., Adriani A., Amin S. (2020). Successful diagnosis and management of tuberculosis verrucosa cutis using antituberculosis therapy trial approach. Pan Afr. Med. J..

[12] Sudarshan R., Nayak K., Kumar P., Kadilkar U. (2016). Rare Case of Multifocal Cutaneous Tuberculosis Verrucosa Cutis: Posing Clinical and Histopathological Diagnostic Dilemma. J. Adv. Med. Med. Res..

[13] Sehgal V.N., Verma P., Bhattacharya S.N., Sharma S., Singh N. (2017). Multifocal Tuberculosis Verrucosa Cutis: A Manifestation Extraordinary of Reactivation Secondary Tuberculosis. Skinmed.

[14] Rani S., Bansal P., Ahuja A., Agrawal D. (2020). Varied Presentation of Cutaneous Tuberculosis in a Patient. Indian J. Derm. Diagn Dermatol..

[15] Prasad P.V.S., Ambujam S., Paul E.K., Krishnasamy B., Veliath A.J. (2002). Multifocal Tuberculous Verrucosa Cutis: An Unusual Clinical Presentation. Indian J. Tuberc..

[16] Padmaprasad M.K. (2013). Case report: Tuberculosis verrucosa cutis. J. Evol. Med. Dent. Sci..

[17] Vora R.V., Diwan N.G., Rathod K.J. (2016). Tuberculosis verrucosa cutis with multifocal involvement. Indian Dermatol. Online J..

[18] van Zyl L., du Plessis J., Viljoen J. (2015). Cutaneous tuberculosis overview and current treatment regimens. Tuberculosis.

[19] dos Santos J.B., Figueiredo A.R., Ferraz C.E., de Oliveira M.H., da Silva P.G., de Medeiros V.L.S. (2014). Cutaneous tuberculosis: Diagnosis, histopathology and treatment—Part II. An. Bras. Dermatol..

[20] Cutaneous Tuberculosis: A Clinico-Morphological Study—PubMed. https://pubmed.ncbi.nlm.nih.gov/27688538/.

[21] Frankel A., Penrose C., Emer J. (2009). Cutaneous tuberculosis: A practical case report and review for the dermatologist. J. Clin. Aesthet. Dermatol..

